# Clinical characteristics and outcomes of COVID-19 patients admitted to the intensive care unit during the first year of the pandemic in Brazil: a single center retrospective cohort study

**DOI:** 10.31744/einstein_journal/2021AO6739

**Published:** 2021-11-19

**Authors:** Thiago Domingos Corrêa, Thais Dias Midega, Karina Tavares Timenetsky, Ricardo Luiz Cordioli, Carmen Silvia Valente Barbas, Moacyr Silva, Bruno de Arruda Bravim, Bruno Caldin Silva, Gustavo Faissol Janot de Matos, Ricardo Kenji Nawa, Fabrício Rodrigues Torres de Carvalho, Verônica Neves Fialho Queiroz, Roberto Rabello, Felipe Maia de Toledo Piza, Adriano José Pereira, Marcele Liliane Pesavento, Raquel Afonso Caserta Eid, Bento Fortunato Cardoso dos Santos, Andreia Pardini, Vanessa Damázio Teich, Claudia Regina Laselva, Miguel Cendoroglo, Sidney Klajner, Leonardo José Rolim Ferraz

**Affiliations:** 1 Hospital Israelita Albert Einstein São Paulo SP Brazil Hospital Israelita Albert Einstein, São Paulo, SP, Brazil.

**Keywords:** Coronavirus, COVID-19, Coronavirus infections, SARS-CoV-2, Betacoronavirus, Intensive care units, Respiration, artificial, Noninvasive ventilation, Extracorporeal membrane oxygenation, Critical care outcomes, Mortality

## Abstract

**Objective:**

To describe clinical characteristics, resource use, outcomes, and to identify predictors of in-hospital mortality of patients with COVID-19 admitted to the intensive care unit.

**Methods:**

Retrospective single-center cohort study conducted at a private hospital in São Paulo (SP), Brazil. All consecutive adult (≥18 years) patients admitted to the intensive care unit, between March 4, 2020 and February 28, 2021 were included in this study. Patients were categorized between survivors and non-survivors according to hospital discharge.

**Results:**

During the study period, 1,296 patients [median (interquartile range) age: 66 (53-77) years] with COVID-19 were admitted to the intensive care unit. Out of those, 170 (13.6%) died at hospital (non-survivors) and 1,078 (86.4%) were discharged (survivors). Compared to survivors, non-survivors were older [80 (70-88) *versus* 63 (50-74) years; p<0.001], had a higher Simpliﬁed Acute Physiology Score 3 [59 (54-66) *versus* 47 (42-53) points; p<0.001], and presented comorbidities more frequently. During the intensive care unit stay, 56.6% of patients received noninvasive ventilation, 32.9% received mechanical ventilation, 31.3% used high flow nasal cannula, 11.7% received renal replacement therapy, and 1.5% used extracorporeal membrane oxygenation. Independent predictors of in-hospital mortality included age, Sequential Organ Failure Assessment score, Charlson Comorbidity Index, need for mechanical ventilation, high flow nasal cannula, renal replacement therapy, and extracorporeal membrane oxygenation support.

**Conclusion:**

Patients with severe COVID-19 admitted to the intensive care unit exhibited a considerable morbidity and mortality, demanding substantial organ support, and prolonged intensive care unit and hospital stay.

## INTRODUCTION

Coronavirus disease 2019 (COVID-19) is an emerging infectious disease that was first reported in Wuhan, China, and has subsequently spread worldwide.^(1)^ Although most of the infected patients develop only mild symptoms, approximately 15% of symptomatic patients will require hospitalization,^(2)^ and almost 20% of hospitalized patients will require intensive care unit (ICU) admission due to progression to acute respiratory failure (ARF).^(3,4)^

Advanced age, male sex, obesity, systemic hypertension, *diabetes mellitus*, chronic obstructive pulmonary disease (COPD), and cardiovascular disease are major risk factors for severe COVID-19.^(5-8)^ Critically ill patients with COVID-19 require substantial organ support and prolonged ICU stay.^(9)^ For instance, a systematic review including 16,561 critically ill COVID-19 patients demonstrated approximately 76% of COVID-19 patients admitted to the ICU developed acute respiratory distress syndrome (ARDS), two thirds of patients used mechanical ventilation, and 17% of them required renal replacement therapy (RRT).^(9)^

The first case of COVID-19 in Brazil was confirmed at *Hospital Israelita Albert Einstein* (HIAE), on February 26, 2020.^(2)^ Up to June 2021, more than 16 million cases and 500 thousand deaths due to COVID-19 had been registered in Brazil.^(10)^ Nevertheless, few studies reported on epidemiology, clinical characteristics, resource use, and outcomes of ICU patients with COVID-19, in Brazil.^(11-14)^

## OBJECTIVE

To describe clinical characteristics, resource use, outcomes, and to identify predictors of in-hospital mortality of patients with COVID-19 admitted to the intensive care unit.

## METHODS

### Study design

We performed a single center retrospective cohort study. The study was approved by the Local Ethics Committee at HIAE with waiver of Informed Consent (CAAE: 30797520.6.0000.0071, protocol number: 4.562.815). This study is reported in accordance with the effort Strengthening the Reporting of Observational Studies in Epidemiology (STROBE).^(15)^

### Setting

This study was conducted in a private quaternary care hospital located in São Paulo, SP, Brazil. The HIAE comprises 634 beds. Out of those, 37 were open medical-surgical adult ICU beds and 81 were adult step-down unit beds. During the first year of the COVID-19 pandemic, the total ICU operational capacity was increased, reaching 81 ICU beds designated to support severe COVID-19 patients requiring intensive care.

### Study participants

Consecutive adult (≥18 years) patients admitted to the ICU, from March 4, 2020 to February 28, 2021 and diagnosed with COVID-19 were eligible for inclusion in this study. Laboratory confirmation of severe acute respiratory syndrome coronavirus 2 (SARS-CoV-2) infection was based on positive reverse transcriptase polymerase chain reaction (RT-PCR) assay (Cobas^®^ SARS-CoV-2 Test, Roche Molecular Systems, Branchburg, NJ, United States).^(16)^

### Patient management

Criteria for ICU admission and the institutional protocol for severe SARS-CoV-2 infection management have been published elsewhere.^(17,18)^

### Data collection and study variables

All study data were retrieved from Epimed Monitor system (Epimed Solutions, Rio de Janeiro, Brazil), which is an electronic structured case report form, in which patients’ data are prospectively entered by trained ICU case managers.^(19)^ Collected variables included demographics, comorbidities, Simpliﬁed Acute Physiology Score (SAPS 3)^(20)^ at ICU admission, Sequential Organ Failure Assessment (SOFA)^(21)^ at ICU admission, Charlson Comorbidity Index,^(22)^ modified frailty index (MFI),^(23)^ resource use and organ support (vasopressors, noninvasive ventilation, high flow nasal cannula – HFNC –, mechanical ventilation, and extracorporeal membrane oxygenation – ECMO), during ICU stay, destination at hospital discharge, ICU and hospital length of stay, and ICU and in-hospital mortality.

### Statistical analysis

Categorical variables are presented as absolute and relative frequencies. Continuous variables are presented as median with interquartile range (IQR). Normality was assessed by the Kolmogorov-Smirnov test.

Comparisons were made between survivors and non-survivors, based on in-hospital mortality. Categorical variables were compared using the χ^2^ test or Fisher exact test, when appropriate. Continuous variables were compared using independent *t* test, or Mann-Whitney U test in case of non-normal distribution. Survival at day 28 of pooled patients, and survival stratified according to the use of mechanical ventilation, RRT and ECMO, were analyzed by means of the Kaplan-Meier method. Patients discharged from hospital before 28 days were considered alive at day 28.

Univariable logistic regression analysis was performed to identify which predictors were associated with in-hospital mortality. Multivariable logistic regression analyses with backward elimination procedure, including all the predictors showing a p-value <0.10 in the univariable analysis, were undertaken to obtain adjusted odds ratio (OR), along with 95% confidence interval (95%CI), and to define which variables were independently associated with in-hospital mortality. We tested the linearity assumption for continuous variables included in logistic regression models by analyzing the interaction between each predictor and its own log (natural log transformation).^(24)^ Whenever the linearity assumption was violated, continuous variables were categorized. Final multivariable logistic regression model discrimination (area under a Receiver Operating Characteristic Curve – AUC) and calibration (Hosmer-Lemeshow χ^2^ statistic) were reported.^(25)^

Two-tailed tests were used, and considered statistically significant when p<0.05. All analyses were performed using IBM (SPSS) for Macintosh, version 27.0., and GraphPad Prism version 9.0 (GraphPad Software, California, United States) was used for graph plotting.

## RESULTS

### Cohort included

Between March 4, 2020 and February 28, 2021, 1,296 patients with laboratory confirmed COVID-19 were admitted to the ICU. Out of those, 170 (13.6%) died at hospital (non-survivors) and 1.078 (86.4%) were discharged alive from the hospital (survivors). By the time data were extracted (March 9, 2021), 48 patients were still hospitalized. Baseline characteristics of patients are shown in table 1.

**Table 1:** Baseline characteristics of studied patients according to in-hospital mortality

Characteristics		All patients 1,296 (100%)*	Survivors 1,078/1,248 (86.4%)	Non-survivors 170/1,248 (13.6%)	p value
Age, years		66 (53-77)	63 (50-74)	80 (70-88)	<0.001†
Men		862/1,296 (66.5)	718/1,078 (66.6)	109/170 (64.1)	0.582‡
SAPS 3		49 (42-56)	47 (42-53)	59 (54-66)	<0.001†
SOFA		1 (0-5)	1 (0-3)	6 (4-9)	<0.001†
CCI		1 (0-1)	0 (0-1)	2 (1-3)	<0.001†
MFI, points		1 (0-2)	1 (0-2)	2 (1-3)	<0.001†
Comorbidities					
	Hypertension	607/1,022 (59.4)	484/814 (59.5)	97/164 (59.1)	1.000‡
	*Diabetes mellitus*	366/1,022 (35.8)	286/814 (35.1)	64/164 (39.0)	0.391‡
	Obesity	320/1,043 (30.7)	265/857 (30.9)	47/146 (32.2)	0.834‡
	Asthma	84/1,022 (8.2)	70/814 (8.6)	10/164 (6.1)	0.363‡
	Cancer	80/1,022 (7.8)	55/814 (6.8)	21/164 (12.8)	0.013‡
	Congestive heart failure	74/1,022 (7.2)	46/814 (5.7)	25/164 (15.2)	<0.001‡
	COPD	74/1,022 (7.2)	52/814 (6.4)	17/164 (10.4)	0.099‡
	Chronic kidney disease	56/1,022 (5.5)	32/814 (3.9)	17/164 (10.4)	0.001‡
	Chronic kidney disease requiring RRT	16/1,022 (1.6)	9/814 (1.1)	7/164 (4.3)	0.010‡
	Hematologic cancer	40/1,022 (3.9)	27/814 (3.3)	11/164 (6.7)	0.068‡
	Metastatic cancer	24/1,022 (2.3)	15/814 (1.8)	7/164 (4.3)	0.105‡
Days from hospital to ICU admission		1 (0-2)	1 (0-2)	0 (0-1)	0.096†
Support at ICU admission					
	Noninvasive ventilation	267/1,296 (20.6)	218/1,078 (20.2)	34/170 (20.0)	1.000‡
	Mechanical ventilation	111/1,296 (8.6)	82/1,078 (7.6)	20/170 (11.8)	0.091‡
	Vasopressors	83/1,296 (6.4)	53/1,078 (4.9)	20/170 (11.8)	<0.001‡
	Renal replacement therapy	4/1,296 (0.3)	1/1,078 (0.1)	2/170 (1.2)	0.050‡

Results expressed as median (interquartile range) or n/n total (%).

* By the time data was extracted (March, 9, 2021), 48 patients were still hospitalized; p-values were calculated using

† Mann-Whitney U test

‡ χ^2^ test.

SAPS 3: Simplified Acute Physiology Score 3; SOFA: Sequential Organ Failure Assessment; CCI: Charlson Comorbidity Index; MFI: modified frailty index; COPD: chronic obstructive pulmonary disease; RRT: renal replacement therapy; ICU: intensive care unit.

The median (IQR) age of pooled patients was 66 (53 to 77) years, 66.5% were male, and the median (IQR) SAPS 3 was 49 (42 to 56) points. The most common comorbidities were hypertension (59.4%), *diabetes mellitus* (35.8%), and obesity (30.7%). At ICU admission, 20.6% of patients were receiving noninvasive ventilation, 8.6% were under mechanical ventilation, and 6.4% were using vasopressors.

Compared to survivors, non-survivors were older [80 (70-88) *versus* 63 (50-74) years; p<0.001], had a higher SAPS 3 [59 (54-66) *versus* 47 (42-53) points; p<0.001] and a higher SOFA [6 (4-9) *versus* 1 (0-3) points; p<0.001] at ICU admission. Cancer, congestive heart failure, chronic kidney disease requiring and not requiring RRT were more frequent in non-survivors compared to survivors.

### Resource use

During the ICU stay, 56.6% of patients received noninvasive ventilation, 32.9% of patients were mechanically ventilated; 31.3% used HFNC, 11.7% received RRT, and 1.5% received ECMO support. The median (IQR) duration of mechanical ventilation in pooled patients was 11 (6 to 24) days (Table 2).

**Table 2:** Resource use

Resource		All patients 1,296 (100%)*	Survivors 1,078/1,248 (86.4%)	Non-survivors 170/1,248 (13.6%)	p value
Support during ICU stay					
	Noninvasive ventilation	733/1,296 (56.6)	602/1,078 (55.8)	104/170 (61.2)	0.222†
	Mechanical ventilation	426/1,296 (32.9)	257/1,078 (23.8)	133/170 (78.2)	<0.001†
	Vasopressors	418/1,296 (32.3)	254/1,078 (23.6)	128/170 (75.3)	<0.001†
	High flow nasal cannula	406/1,296 (31.3)	308/1,078 (28.6)	72/170 (42.4)	<0.001†
	Renal replacement therapy	151/1,296 (11.7)	59/1,078 (5.5)	76/170 (44.7)	<0.001†
	ECMO	20/1,296 (1.5)	6/1,078 (0.6)	11/170 (6.5)	<0.001‡
Tracheostomy		84/1,296 (6.5)	45/1,078 (4.2)	29/170 (17.1)	<0.001†
MV duration (days)		11 (6-24)	9 (5-15)	17 (10-38)	<0.001§

Results expressed as median (interquartile range) or n/n total (%).

* By the time data was extracted (March 9, 2021), 48 patients were still hospitalized; p-values were calculated using

† χ^2^ test

‡ Fisher exact test or

§ Mann-Whitney U test.

ICU: intensive care unit; ECMO: extracorporeal membrane oxygenation; MV: mechanical ventilation.

Mechanical ventilation, HFNC, vasopressors, RRT, and ECMO were more frequently used in non-survivors compared to survivors. The median (IQR) days on mechanical ventilation was higher in non-survivors compared to survivors [17 (10-38) *versus* 9 (5-15) days; p<0.001].

### Clinical outcomes

#### Pooled patients

**Figure 1:**
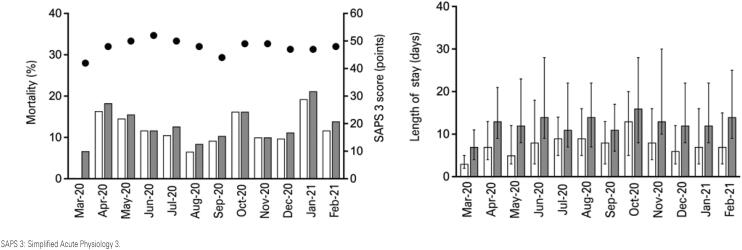
Monthly length of intensive care unit and hospital stay, intensive care unit and in-hospital mortality, and simplified acute physiology score 3 from March 2020 to February 2021

**Figure 2:**
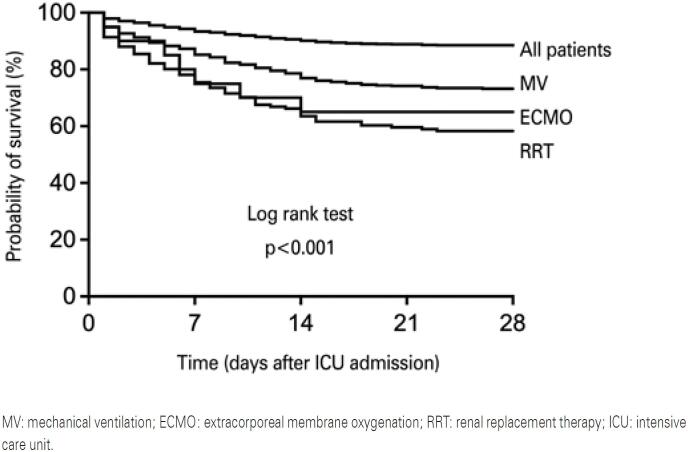
Cumulative survival at day 28 of pooled patients and according to the need for organ support

Intensive care unit and in-hospital mortality of pooled patients was, respectively, 11.7% (151 of 1,296 patients) and 13.6% (170 of 1,248 patients) (Table 3). Monthly ICU and in-hospital mortality between March 2020 and February 2021 is shown in figure 1. Cumulative survival at day 28 of pooled patients and survival stratified according to the use of mechanical ventilation, RRT, and ECMO are shown in figure 2.

**Table 3:** Clinical outcomes

Outcomes		All patients 1,296 (100%)*	Survivors 1,078/1,248 (86.4%)	Non-survivors 170/1,248 (13.6%)	p value
Destination at hospital discharge					<0.001†
	Home	1,050/1,296 (84.1)	1,050/1,078 (97.4)	0/170 (0.0)	
	Home care	18/1,296 (1.4)	18/1,078 (1.7)	0/170 (0.0)	
	Transfer to another hospital	10/1,296 (0.8)	10/1,078 (0.9)	0/170 (0.0)	
	Palliative care	47/1,296 (3.6)	3/1,078 (0.3)	44/170 (25.9)	<0.001†
ICU length of stay, days		7 (4-16)	7 (3-13)	15 (9-29)	<0.001‡
Hospital length of stay, days		13 (8-23)	12 (8-21)	19 (12-34)	<0.001‡
According to the use of MV*					
Patients who received MV		426 (100.0)	257/390 (65.9)	133/390 (34.1)§	
	ICU length of stay, days	20 (13-32)§	19 (13-30)§	19 (12-33)§	0.892‡
	Hospital length of stay, days	27 (17-41)§	28 (18-41)§	24 (14-41)§	0.021‡
Patients who did not receive MV		870 (100.0)	821/858 (95.7)	37/858 (4.3)	
	ICU length of stay, days	5 (2-8)	5 (2-8)	7 (3-11)	0.058‡
	Hospital length of stay (days)	10 (7-15)	10 (7-15)	10.0 (6-15)	0.850‡
According to the use of RRT*					
	Patients who received RRT	151 (100.0)	59/135 (43.7)	76/135 (56.3)¶	
	ICU length of stay, days	27 (15-40)¶	25 (17-37)¶	26 (13-40)¶	0.688‡
	Hospital length of stay, days	33 (22-55)¶	35 (28-61)¶	30 (16-49)¶	0.024‡
Patients who did not receive RRT		1,145 (100.0)	1,019/1,113 (91.6)	94/1,113 (8.4)	
	ICU length of stay, days	7 (3-13)	6 (3-11)	13 (6-17)	<0.001‡
	Hospital length of stay, days	12 (8-19)	11 (8-19)	15 (9-24)	0.009‡
According to the use of ECMO*					
	Patients who received ECMO	20 (100.0)	6/17 (35.3)	11/17 (64.7)&	
	ICU length of stay, days	32 (24-59)&	37 (23-76)&	29 (10-55)#	0.687‡
	Hospital length of stay, days	48 (29-70)&	49 (33-98)&	31 (12-70)	0.365‡
Patients who did not receive ECMO		1,276 (100.0)	1,072/1,231 (87.1)	159/1,231 (12.9)	
	ICU length of stay, days	7 (3-15)	7 (3-13)	15 (9-28)	<0.001‡
	Hospital length of stay, days	12 (8-23)	12 (8-21)	18 (12-34)	<0.001‡

Results expressed as n/n total (%) or median (interquartile range).

* By the time data was extracted (March 9, 2021), 48 patients were still hospitalized. Out of those, 36 patients were receiving MV, 16 were receiving RRT, and 3 were receiving ECMO; p-values were calculated using

† χ^2^ test

‡ Mann-Whitney U test

§ p<0.001 *versus* patients who did not receive MV

¶ p<0.001 *versus* patients who did not receive RRT

& p<0.001 *versus* patients who did not receive ECMO

# p=0.039 *versus* patients who did not receive ECMO.

ICU: intensive care unit; MV: mechanical ventilation; RRT: renal replacement therapy; ECMO: extracorporeal membrane oxygenation.

The median IQR length of ICU and hospital stay was, respectively, 7 (4 to 16) days and 13 (8 to 23) days. Compared to survivors, non-survivors exhibited a higher length of ICU [15 (9-29) *versus* 7 (3-13) days; p<0.001] and hospital stay [19 (12-34) *versus* 12 (8-21) days; p<0.001] (Table 3).

#### Mechanically ventilated patients

Mechanically ventilated patients exhibited a higher in-hospital mortality compared to patients who did not receive mechanical ventilation (34.1% *versus* 4.3%; unadjusted OR: 11.5; 95%CI: 7.8-17.0; p<0.001). The length of ICU [20 (13-32) *versus* 5 (2-8) days; p<0.001] and hospital stay [27 (17-41) *versus* 10 (7-15) days; p<0.001] was higher in patients who received mechanical ventilation compared to non-mechanically ventilated patients.

#### Patients who received renal replacement therapy

Patients submitted to RRT showed a higher in-hospital mortality compared to patients who did not receive RRT (56.3% *versus* 8.4%; unadjusted OR: 14.0; 95%CI: 9.4-20.8; p<0.001). Mechanical ventilation was used in 143 of 151 (94.7%) patients who received RRT. The length of ICU [27 (15-40) *versus* 7 (3-13) days; p<0.001] and hospital stay [33 (22-55) *versus* 12 (8-19) days; p<0.001] was higher in patients who received RRT than in patients who did not receive RRT.

#### Patients who received extracorporeal membrane oxygenation

Patients who received ECMO exhibited a higher in-hospital mortality compared to patients who did not receive ECMO [64.7% *versus* 12.9%; unadjusted OR: 12.4; 95%CI: 4.5-33.9; p<0.001) (Table 3). All patients who received ECMO support received mechanical ventilation, and 13 out of 20 (65.0%) patients also used RRT. The length of ICU [32 (24-59) *versus* 7 (3-15) days; p<0.001] and hospital stay [48 (29-70) *versus* 12 (8-23) days; p<0.001] was higher in patients who received ECMO than in patients who did not receive ECMO support.

#### Predictors of in-hospital mortality

Univariable analysis of factors associated with in-hospital death is depicted in table 4. After adjusting for confounders, independent predictors of in-hospital mortality included age (OR: 1.08; 95%CI: 1.06-1.10; p<0.001); SOFA (OR: 1.18; 95%CI: 1.08-1.29; p<0.001); Charlson Comorbidity Index (OR: 1.28; 95%CI: 1.15-1.43; p<0.001); the need for mechanical ventilation (OR: 4.45; 95%CI: 2.43-8.16; p<0.001); the need for HFNC (OR: 1.64; 95%CI: 1.04-2.58; p=0.033); RRT (OR: 3.42; 95%CI: 1.96-5.98; p<0.001) and ECMO support (OR: 8.18; 95%CI: 2.48-27.05; p<0.001) (Table 4). The final multivariable model had an area under the ROC curve (95%CI) of 0.93 (0.91-0.94) and a Hosmer-Lemeshow χ^2^ of 8.844 (p=0.356).

**Table 4:** Univariable and multivariable logistic regression analyses addressing risk factors for in-hospital mortality

Predictors		Type of analysis Univariable analysis			Multivariable analysis		
		OR	95%CI	p value	OR	95%CI	p value
Age, years		1.07	1.06-1.08	<0.001	1.08	1.06-1.10	<0.001
SAPS 3*							
	≤42	Reference					
	43-49	2.93	1.04-8.23	0.041			
	50-55	9.77	3.75-25.46	<0.001			
	≥56	41.80	16.79-104.07	<0.001			
SOFA		1.46	1.38-1.54	<0.001	1.18	1.08-1.29	<0.001
Charlson Comorbidity Index		1.39	1.29-1.51	<0.001	1.28	1.15-1.43	<0.001
MFI		1.72	1.53-1.95	<0.001			
Comorbidity							
	Cancer	2.03	1.19-3.46	0.009			
	Congestive heart failure	3.00	1.79-5.05	<0.001			
	COPD	1.70	0.95-3.01	0.072			
	Chronic kidney disease	2.83	1.53-5.22	<0.001			
	Chronic kidney disease requiring RRT	3.99	1.46-10.87	0.007			
	Hematologic cancer	2.10	1.02-4.31	0.045			
	Metastatic cancer	2.38	0.95-5.92	0.063			
Days from hospital to ICU admission		1.00	0.99-1.02	0.534			
AKI at ICU admission		3.41	1.86-6.26	<0.001			
Support during ICU stay							
	Mechanical ventilation	11.48	7.77-16.97	<0.001	4.45	2.43-8.16	<0.001
	Vasopressors	9.89	6.79-14.40	<0.001			
	High flow nasal cannula	1.84	1.32-2.56	<0.001	1.64	1.04-2.58	0.033
	Renal replacement therapy	11.96	9.36-20.84	<0.001	3.42	1.96-5.98	<0.001
	ECMO	12.36	4.51-33.89	<0.001	8.18	2.48-27.05	<0.001

* SAPS 3 was categorized according to percentiles, since linearity assumption was violated. The multivariable model had an area under the Receiver Operating Characteristic curve (95%CI) of 0.93 (0.91-0.94) and a Hosmer-Lemeshow χ^2^ of 8.844 (p=0.356).

OR: odds ratio; 95%CI: 95% confidence interval; SAPS 3: Simplified Acute Physiology Score 3; SOFA score: Sequential Organ Failure Assessment score; MFI: modified frailty index; COPD: chronic obstructive pulmonary disease; RRT: renal replacement therapy; ICU: intensive care unit; AKI: acute kidney injury according; ECMO: extracorporeal membrane oxygenation.

## DISCUSSION

In this retrospective single center cohort study, we found that one in seven patients admitted to the ICU due to severe COVID-19 infection died at the hospital. Non-survivors were older, sicker, in accordance with SAPS 3 and SOFA, presented more comorbidities, such as cancer, congestive heart failure or chronic kidney disease, and had a longer ICU and hospital length of stay compared to survivors. Finally, increased age, higher SOFA score and Charlson Comorbidity Index, need for mechanical ventilation, HFNC, RRT, and ECMO were independent predictors of in-hospital mortality.

The association between advanced age and increased risk of death in patients infected with SARS-CoV-2 has been reported by different authors.^(3,26-28)^ Moreover, the association between the presence of comorbidities and the severity of COVID-19 was evidenced in several studies.^(9,26,29,30)^ For instance, COVID-19 patients with hypertension, cardiocerebrovascular diseases, and *diabetes mellitus* were at higher risk of developing severe symptoms, and requiring ICU admission than patients without these comorbidities.^(30)^ Furthermore, we observed that locoregional cancer diagnosis was more prevalent in deceased patients in comparison with survivors. Indeed, it was demonstrated in a large case-control study that cancer patients with COVID-19 exhibited an increased risk of worse clinical outcomes.^(31)^ Although increased mortality has also been reported in patients with hematological cancer,^(32)^ we did not observe this association in our study.

Interestingly, in our cohort we found lower in-hospital mortality both among patients admitted to the ICU and those requiring mechanical ventilation support, compared to previous studies conducted in Brazil^(14)^ and in other countries.^(6,33,34)^ In a cohort study involving 254,288 patients hospitalized with COVID-19 in Brazil, Ranzani *et al.,* demonstrated a hospital mortality rate of approximately 60% among patients admitted to the ICU and a rate of roughly 80% among patients who received mechanical ventilation.^(14)^ In a meta-analysis comprising 28 studies and 12,437 patients admitted to the ICU with COVID-19 worldwide, the reported ICU and mechanically ventilated mortality were, respectively, 28.3% and 43%.^(4)^

The discrepancy between the observed mortality rate in the present study and in other series may be explained by different hospital characteristics (private *versus* public); adopted thresholds for hospitalization and/or ICU admission; availability of resources, such as ICU beds, and limited offer of advanced respiratory support outside an ICU, or in ICUs but with resource constraints, characteristics of the ICU staff; ventilation strategies, such as the use of noninvasive ventilation and HFNC, and strategies for extra-pulmonary organ support. Furthermore, despite the different moments of COVID-19 pandemic in Brazil, the observed monthly stability on outcomes (length of stay and mortality) of COVID-19 patients reflects the hospital and ICU organization to provide the best quality of care to patients.

Patients with acute kidney injury requiring RRT had a 14-fold increase in the *odds* for in-hospital death, compared with COVID-19 patients who did not receive RRT. This observation is consistent with other studies, in which a significant association between acute kidney injury and increased risk of death was reported in patients with severe COVID-19.^(4,35,36)^ Additionally, we found that almost half of the deceased patients underwent RRT, which is also similar to other studies.^(34,37,38)^ The assessment of risk factors for the development of acute kidney injury, and use of RRT in the first 207 critically ill COVID-19 patients admitted to our ICU, were reported elsewhere.^(39)^

The highest in-hospital mortality rate in our study was observed in the subgroup of patients who received ECMO (approximately 65%). Reported mortality for COVID-19 patients submitted to veno-venous ECMO varied widely.^(40)^ For instance, the reported in-hospital death at 90 days after ECMO initiation in 1,035 patients from the Extracorporeal Life Support Organization (ELSO) registry was 37%.^(41)^ Nevertheless, approximately one third of patients included in this study were still hospitalized, or had been discharged to another hospital or to a long-term acute care center at the time of outcome measurement.^(41)^ Therefore, the crude mortality for COVID-19 patients who receive ECMO may be underestimated.

Our study has limitations. First, it was performed in a single ICU located in a private quaternary care hospital in Brazil. Therefore, our results may not be generalizable to other ICUs in Brazil or in other developing countries, since healthcare systems and patient characteristics may vary substantially from our cohort. Second, we did not collect detailed data on noninvasive or on ventilator management strategies. It is well established that ventilatory support for ARF patients has a major impact on outcomes.^(42,43)^ Third, we assumed that patients discharged from hospital before 28 days were still alive at day 28. Nevertheless, discharged patients might have been readmitted elsewhere or could have died after discharge. Finally, we did not assess whether the new SARS-CoV-2 variants affected outcomes in our cohort of patients compared to previously circulating variants in Brazil.^(44,45)^

## CONCLUSION

Patients with severe COVID-19 admitted to the intensive care unit had considerable morbidity and mortality, requiring substantial organ support, and a prolonged intensive care unit and hospital stay. The volume and severity of COVID-19 patients requiring intensive care unit admission are a great burden for the Brazilian health care system. Therefore, the results of this study may be of interest to be used as benchmark, or to support decisions concerning delivery of healthcare services and prognosis for COVID-19 patients demanding intensive care.
