# Supplementary material for: Clinical characteristics and outcomes of COVID-19 patients admitted to the intensive care unit during the first year of the pandemic in Brazil: a single center retrospective cohort study
Source: Einstein (Sao Paulo). 2021 Nov 19;19:eAO6739. doi: 10.31744/einstein_journal/2021AO6739 (PMC8664289; doi:10.31744/einstein_journal/2021AO6739)
Supplement: Translation

# Translation: Características clínicas e desfechos de pacientes com COVID-19 admitidos em unidade de terapia intensiva durante o primeiro ano de pandemia no Brasil: um estudo de coorte retrospectivo em centro único

**Authors:** Thiago Domingos Corrêa, Thais Dias Midega, Karina Tavares Timenetsky, Ricardo Luiz Cordioli, Carmen Silvia Valente Barbas, Moacyr Silva, Bruno de Arruda Bravim, Bruno Caldin Silva, Gustavo Faissol Janot de Matos, Ricardo Kenji Nawa, Fabrício Rodrigues Torres de Carvalho, Verônica Neves Fialho Queiroz, Roberto Rabello, Felipe Maia de Toledo Piza, Adriano José Pereira, Marcele Liliane Pesavento, Raquel Afonso Caserta Eid, Bento Fortunato Cardoso dos Santos, Andreia Pardini, Vanessa Damázio Teich, Claudia Regina Laselva, Miguel Cendoroglo, Sidney Klajner, Leonardo José Rolim Ferraz

**Affiliations:** 1 São Paulo SP Brasil Hospital Israelita Albert Einstein, São Paulo, SP, Brasil.

**Keywords:** Coronavírus, COVID-19, Infecções por coronavírus, SARS-CoV-2, Betacoronavírus, Unidades de terapia intensiva, Respiração artificial, Ventilação não invasiva, Oxigenação por membrana extracorpórea, Resultados de cuidados críticos, Mortalidade

## Abstract

**Objetivo:**

Descrever características clínicas, uso de recursos e desfechos e identificar preditores de mortalidade intra-hospitalar de pacientes com COVID-19 admitidos na unidade de terapia intensiva.

**Métodos:**

Estudo de coorte retrospectivo, em centro único, realizado em um hospital privado localizado em São Paulo (SP). Pacientes adultos (≥18 anos) admitidos consecutivamente na unidade de terapia intensiva, entre 4 de março de 2020 a 28 de fevereiro de 2021, foram incluídos neste estudo. Os pacientes foram classificados como sobreviventes e não sobreviventes, de acordo com a alta hospitalar.

**Resultados:**

Durante o período do estudo, 1.296 pacientes [mediana (intervalo interquartil) de idade: 66 (53-77) anos] com COVID-19 foram admitidos na unidade de terapia intensiva. Destes, 170 (13,6%) pacientes morreram no hospital (não sobreviventes), e 1.078 (86,4%) receberam alta hospitalar (sobreviventes). Comparados aos sobreviventes, os não sobreviventes eram mais idosos [80 (70-88) *versus* 63 (50-74) anos; p<0,001], apresentavam pontuação mais alta no sistema prognóstico *Simpliﬁed Acute Physiology Score* *3* [59 (54-66) *versus* 47 (42-53); pontos p<0,001] e tinham mais comorbidades. Durante a internação na unidade de terapia intensiva, 56,6% dos pacientes usaram ventilação não invasiva, 32,9% usaram ventilação mecânica invasiva, 31,3% usaram cateter nasal de alto fluxo, 11,7% foram submetidos à terapia renal substitutiva, e 1,5% usou oxigenação por membrana extracorpórea. Os preditores independentes de mortalidade intra-hospitalar foram idade, *Sequential Organ Failure Assessment*, Índice de Comorbidade de Charlson, necessidade de ventilação mecânica, uso de cateter nasal de alto fluxo, uso de terapia renal substitutiva e suporte por oxigenação por membrana extracorpórea.

**Conclusão:**

Pacientes com quadros graves da COVID-19 admitidos na unidade de terapia intensiva apresentaram considerável mortalidade e morbidade, com alta demanda de terapia de suporte e internação prolongada em unidade de terapia intensiva e hospitalar.

## INTRODUÇÃO

A doença pelo coronavírus 2019 (COVID-19) é uma doença infecciosa emergente que foi registrada pela primeira vez em Wuhan, na China, e, posteriormente, propagou-se para o resto do mundo.^(1)^ Embora a maioria dos indivíduos infectados apresente somente sintomas leves, aproximadamente 15% dos pacientes sintomáticos precisam ser hospitalizados,^(2)^ e quase 20% dos hospitalizados precisam de internação em unidade de terapia intensiva (UTI), devido à progressão para insuficiência respiratória aguda (IRA).^(3,4)^

Idade avançada, sexo masculino, obesidade, hipertensão sistêmica, *diabetes mellitus*, doença pulmonar obstrutiva crônica (DPOC) e doença cardiovascular são os principais fatores de risco para quadro grave da COVID-19.^(5-8)^ Pacientes com quadro crítico da COVID-19 necessitam de amplo suporte invasivo e internação prolongada em UTI.^(9)^ Por exemplo: uma revisão sistemática incluindo 16.561 pacientes críticos da COVID-19 demonstrou que aproximadamente 76% dos pacientes com COVID-19 admitidos em UTI apresentaram síndrome do desconforto respiratório agudo (SDRA), dois terços receberam ventilação mecânica, e 17% receberam terapia renal substitutiva (TRS).^(9)^

O primeiro caso da COVID-19 no Brasil foi confirmado em 26 de fevereiro de 2020, no Hospital Israelita Albert Einstein (HIAE).^(2)^ Até junho de 2021, mais de 16 milhões de casos e 500 mil mortes atribuídas à COVID-19 foram registradas no Brasil.^(10)^ No entanto, há poucos estudos sobre epidemiologia, características clínicas, uso de recursos e desfechos dos pacientes da COVID-19 admitidos em UTI, no Brasil.^(11-14)^

## OBJETIVO

Descrever características clínicas, uso de recursos e desfechos e identificar preditores de mortalidade intra-hospitalar de pacientes com COVID-19 admitidos na unidade de terapia intensiva.

## MÉTODOS

### Desenho do estudo

Foi realizado um estudo de coorte retrospectivo em um único centro. O estudo foi aprovado pelo Comitê de Ética do HIAE com isenção da exigência de consentimento livre e esclarecido (CAAE: 30797520.6.0000.0071, parecer número: 4562815). Este estudo foi reportado de acordo com a declaração da iniciativa *Strengthening the Reporting of Observational Studies in Epidemiology* (STROBE).^(15)^

### Local

Este estudo foi realizado em um hospital privado de atenção quaternária localizado em São Paulo (SP). No momento da pesquisa, o HIAE contava com 634 leitos. Destes, 37 eram leitos abertos médico-cirúrgicos de cuidados intensivos para adultos, e 81 eram leitos de cuidados semi-intensivos. Durante o primeiro ano da pandemia da COVID-19, a capacidade operacional total de UTI foi ampliada, chegando a 81 leitos de UTI para pacientes com quadro grave da COVID-19 com necessidade de cuidados intensivos.

### Participantes do estudo

Pacientes adultos (≥18 anos) consecutivos admitidos na UTI, de 4 de março de 2020 a 28 de fevereiro de 2021, diagnosticados com COVID-19, qualificaram-se para inclusão neste estudo. A confirmação laboratorial de infecção pelo coronavírus da síndrome respiratória aguda grave 2 (SARS-CoV-2) foi baseada em resultado positivo no teste de reação em cadeia da polimerase via transcriptase reversa (RT-PCR; Cobas^®^ SARS-CoV-2, Roche Molecular Systems, Branchburg, NJ, Estados Unidos).^(16)^

### Manejo dos pacientes

Os critérios para admissão na UTI e o protocolo institucional de manejo de quadro grave de infecção pelo SARS-CoV-2 foram descritos em outras publicações.^(17,18)^

### Coleta de dados e variáveis do estudo

Todos os dados do estudo foram retirados do sistema Epimed Monitor (Epimed Solutions, Rio de Janeiro, Brasil), uma ficha clínica eletrônica estruturada, na qual os dados dos pacientes são prospectivamente inseridos por gerentes de casos de UTI capacitados para esse fim.^(19)^ As variáveis coletadas incluíram dados demográficos, comorbidades, *Simpliﬁed Acute Physiology Score* (SAPS 3)^(20)^ no momento da admissão na UTI, *Sequential Organ Failure Assessment* (SOFA)^(21)^ no momento da admissão na UTI, Índice de Comorbidade de Charlson,^(22)^ *modified frailty index* (MFI),^(23)^ uso de recursos e terapia de suporte (vasopressores, ventilação não invasiva, cateter nasal de alto fluxo – CNAF –, ventilação mecânica e oxigenação por membrana extracorpórea – ECMO - *extracorporeal membrane oxygenation*), durante a internação em UTI, destino após alta hospitalar, tempo de internação em UTI e hospital e mortalidade em UTI e intra-hospitalar.

### Análise estatística

As variáveis categóricas são apresentadas em frequên-cias absolutas e relativas. Variáveis categóricas são apre-sentadas como mediana com intervalo interquartil (IIQ). A normalidade foi avaliada pelo teste de Kolmogorov-Smirnov.

Comparações foram feitas entre os sobreviventes e não sobreviventes, com base na mortalidade hospitalar. Variáveis categóricas foram comparadas por meio do teste do χ^2^ ou teste exato de Fisher, quando apropriado. Variáveis contínuas foram comparadas usando o teste *t* independente, ou o teste U de Mann-Whitney em caso de distribuição não normal. A sobrevida aos 28 dias de todos os pacientes do estudo e a sobrevida estratificada de acordo com o uso de ventilação mecânica, TRS e ECMO foram analisadas por meio do método Kaplan-Meier. Os pacientes que receberam alta hospitalar antes de 28 dias foram considerados como se ainda estivessem vivos no dia 28.

Análise de regressão logística univariada foi realizada para identificar quais preditores tiveram associação com a mortalidade hospitalar. A análises de regressão logística multivariada com procedimento de eliminação reversa, incluindo todos os preditores com p<0,10 na análise univariada, foi realizada para obtenção da razão de chance (RC) ajustada, bem como do intervalo de confiança de 95% (IC95%), e para definir quais variáveis tiveram associação independente com a mortalidade hospitalar. Testou-se o pressuposto de linearidade das variáveis contínuas incluídas nos modelos de regressão logística, analisando as interações entre cada preditor e seu próprio logaritmo (transformação logarítmica natural).^(24)^ Sempre que o pressuposto de linearidade fosse violado, as variáveis contínuas foram categorizadas. A discriminação final (área sob a curva – ASC – Característica de Operação do Receptor) e a calibração (teste estatístico χ^2^ de Hosmer-Lemeshow) do modelo de regressão logística multivariada foram reportadas.^(25)^

Testes bicaudais foram usados e considerados esta-tisticamente significativos quando p<0,05. Todas as aná-lises usaram o programa *IBM* *(SPSS)* para Macintosh, versão 27.0., e o programa *GraphPad Prism*, versão 9.0 (GraphPad Software, California, Estados Unidos), foi usado para plotagem dos gráficos.

## RESULTADOS

### Coorte incluída

Entre 4 de março de 2020 e 28 de fevereiro de 2021, 1.296 pacientes com diagnóstico laboratorial confirmado da COVID-19 foram admitidos na UTI. Destes, 170 (13,6%) morreram no hospital (não sobreviventes), e 1.078 (86,4%) tiveram alta ainda vivos do hospital (sobreviventes). No momento da extração dos dados (9 de março de 2021), 48 pacientes ainda estavam hospitalizados. As características iniciais dos pacientes são apresentadas na tabela 1.

**Tabela 1:** Características iniciais dos pacientes estudados de acordo com a mortalidade hospitalar

Características		Todos os pacientes 1.296 (100%)*	Sobreviventes 1.078/1.248 (86,4%)	Não sobreviventes 170/1.248 (13,6%)	Valor de p
Idade, anos		66 (53-77)	63 (50-74)	80 (70-88)	<0,001†
Homens		862/1.296 (66,5)	718/1.078 (66,6)	109/170 (64,1)	0,582‡
SAPS 3		49 (42-56)	47 (42-53)	59 (54-66)	<0,001†
SOFA		1 (0-5)	1 (0-3)	6 (4-9)	<0,001†
ICC		1 (0-1)	0 (0-1)	2 (1-3)	<0,001†
MFI, pontos		1 (0-2)	1 (0-2)	2 (1-3)	<0,001†
Comorbidities					
	Hipertensão	607/1.022 (59,4)	484/814 (59,5)	97/164 (59,1)	1,000‡
	*Diabetes mellitus*	366/1.022 (35,8)	286/814 (35,1)	64/164 (39,0)	0,391‡
	Obesidade	320/1.043 (30,7)	265/857 (30,9)	47/146 (32,2)	0,834‡
	Asma	84/1.022 (8,2)	70/814 (8,6)	10/164 (6,1)	0,363‡
	Câncer	80/1.022 (7,8)	55/814 (6,8)	21/164 (12,8)	0,013‡
	Insuficiência cardíaca congestiva	74/1.022 (7,2)	46/814 (5,7)	25/164 (15,2)	<0,001‡
	DPOC	74/1.022 (7,2)	52/814 (6,4)	17/164 (10,4)	0,099‡
	Doença renal crônica	56/1.022 (5,5)	32/814 (3,9)	17/164 (10,4)	0,001‡
	Doença renal crônica com necessidade de TRS	16/1.022 (1,6)	9/814 (1,1)	7/164 (4,3)	0,010‡
	Câncer hematológico	40/1.022 (3,9)	27/814 (3,3)	11/164 (6,7)	0,068‡
	Câncer metastático	24/1.022 (2,3)	15/814 (1,8)	7/164 (4,3)	0,105‡
Dias de internação até a admissão em UTI		1 (0-2)	1 (0-2)	0 (0-1)	0,096†
Suporte no momento da admissão na UTI					
	Ventilação não invasiva	267/1.296 (20,6)	218/1.078 (20,2)	34/170 (20,0)	1,000‡
	Ventilação mecânica	111/1.296 (8,6)	82/1.078 (7,6)	20/170 (11,8)	0,091‡
	Vasopressores	83/1.296 (6,4)	53/1.078 (4,9)	20/170 (11,8)	<0,001‡
	Terapia renal substitutiva	4/1.296 (0,3)	1/1.078 (0,1)	2/170 (1,2)	0,050‡

Resultados expressos como mediana (intervalo interquartil) ou n/total n (%).

* No momento da extração dos dados (9 de março de 2021), 48 pacientes ainda estavam hospitalizados; valores de p foram calculados usando o

† teste U de Mann-Whitney

‡ teste do χ^2^.

SAPS 3: *Simplified Acute Physiology Score 3*; SOFA: *Sequential Organ Failure Assessment Score*; ICC: Índice de Comorbidade de Charlson; MFI: *modified frailty index*; DPOC: doença pulmonar obstrutiva crônica; TRS: terapia renal substitutiva; UTI: unidade de terapia intensiva.

A mediana IIQ de idade de todos os pacientes foi de 66 (53 a 77) anos, 66,5% eram homens e a mediana IIQ do SAPS 3 foi de 49 (42 a 56) pontos. As comorbidades mais comuns foram hipertensão (59,4%), *diabetes mellitus* (35,8%) e obesidade (30,7%). No momento da admissão na UTI, 20,6% dos pacientes estavam usando ventilação não invasiva, 8,6% ventilação mecânica e 6,4% vasopressores.

Comparados aos sobreviventes, os não sobreviventes eram mais idosos [80 (70-88) *versus* 63 (50-74) anos; p<0,001], tinham maior SAPS 3 [59 (54-66) *versus* 47 (42-53) pontos; p<0,001] e maior SOFA [6 (4-9) *versus* 1 (0-3) pontos; p<0,001] no momento da admissão na UTI. Câncer, insuficiência cardíaca congestiva e doença renal crônica com ou sem necessidade de TRS foram mais frequentes em não sobreviventes comparados aos sobreviventes.

### Uso de recursos

Durante a internação em UTI, 56,6% dos pacientes receberam ventilação não invasiva e 32,9% ventilação mecânica; 31,3% usaram CNAF, 11,7% receberam TRS e 1,5% recebeu suporte de ECMO. A mediana IIQ de duração da ventilação mecânica em todos os pacientes do estudo foi de 11 (6 a 24) dias (Tabela 2).

**Tabela 2:** Uso de recursos

Recurso		Todos os pacientes 1.296 (100%)*	Sobreviventes 1.078/1.248 (86,4%)	Não sobreviventes 170/1.248 (13,6%)	Valor de p
Suporte durante internação em UTI					
	Ventilação não invasiva	733/1.296 (56,6)	602/1.078 (55,8)	104/170 (61,2)	0,222†
	Ventilação mecânica	426/1.296 (32,9)	257/1.078 (23,8)	133/170 (78,2)	<0,001†
	Vasopressores	418/1.296 (32,3)	254/1.078 (23,6)	128/170 (75,3)	<0,001†
	Cateter nasal de alto fluxo	406/1.296 (31,3)	308/1.078 (28,6)	72/170 (42,4)	<0,001†
	Terapia renal substitutiva	151/1.296 (11,7)	59/1.078 (5,5)	76/170 (44,7)	<0,001†
	ECMO	20/1.296 (1,5)	6/1.078 (0,6)	11/170 (6,5)	<0,001‡
Traqueostomia		84/1.296 (6,5)	45/1.078 (4,2)	29/170 (17,1)	<0,001†
Duração da VM (dias)		11 (6-24)	9 (5-15)	17 (10-38)	<0,001§

Resultados expressos em n/total n (%) ou mediana (intervalo interquartil).

* No momento da extração dos dados (9 de março de 2021), 48 pacientes ainda estavam hospitalizados; valores p foram calculados usando

† teste do χ^2^

‡ teste exato de Fisher ou

§ teste U de Mann-Whitney.

UTI: unidade de terapia intensiva; ECMO: oxigenação por membrana extracorpórea; VM: ventilação mecânica.

Ventilação mecânica, CNAF, vasopressores, TRS e ECMO foram usados com maior frequência entre os não sobreviventes comparados aos sobreviventes. A mediana IIQ dos dias de ventilação mecânica foi mais alta entre os não sobreviventes comparada à dos sobreviventes [17 (10-38) *versus* 9 (5-15) dias; p<0,001].

### Desfechos clínicos

#### Todos os pacientes

A mortalidade em UTI e hospitalar de todos os pacientes foi de, respectivamente, 11,7% (151 de 1.296 pacientes) e 13,6% (170 de 1.248 pacientes) (Tabela 3). A mortalidade mensal em UTI e hospitalar entre março de 2020 e fevereiro de 2021 é mostrada na figura 1. A sobrevida cumulativa de 28 dias de todos os pacientes e a sobrevida estratificada de acordo com o uso de ventilação mecânica, TRS e ECMO são mostradas na figura 2.

**Tabela 3:** Desfechos clínicos

Desfechos		Todos os pacientes 1.296 (100%)*	Sobreviventes 1.078/1.248 (86,4%)	Não sobreviventes 170/1.248 (13,6%)	Valor de p
Destino após alta hospitalar					<0,001†
	Domicílio	1.050/1.296 (84,1)	1.050/1.078 (97,4)	0/170 (0,0)	
	Assistência domiciliar (*home care*)	18/1.296 (1,4)	18/1.078 (1,7)	0/170 (0,0)	
	Transferência para outro hospital	10/1.296 (0,8)	10/1.078 (0,9)	0/170 (0,0)	
	Cuidados paliativos	47/1.296 (3,6)	3/1.078 (0,3)	44/170 (25,9)	<0,001†
Tempo de internação em UTI, dias		7 (4-16)	7 (3-13)	15 (9-29)	<0,001‡
Tempo de internação hospitalar, dias		13 (8-23)	12 (8-21)	19 (12-34)	<0,001‡
De acordo com o uso de VM*					
Pacientes que receberam VM		426 (100,0)	257/390 (65,9)	133/390 (34,1)§	
	Tempo de internação em UTI, dias	20 (13-32)§	19 (13-30)§	19 (12-33)§	0,892‡
	Tempo de internação hospitalar, dias	27 (17-41)§	28 (18-41)§	24 (14-41)§	0,021‡
Pacientes que não receberam VM		870 (100,0)	821/858 (95,7)	37/858 (4,3)	
	Tempo de internação em UTI, dias	5 (2-8)	5 (2-8)	7 (3-11)	0,058‡
	Tempo de internação hospitalar, dias	10 (7-15)	10 (7-15)	10,0 (6-15)	0,850‡
De acordo com o uso de TRS*					
	Pacientes que receberam TRS	151 (100,0)	59/135 (43,7)	76/135 (56,3)¶	
	Tempo de internação em UTI, dias	27 (15-40)¶	25 (17-37)¶	26 (13-40)¶	0,688‡
	Tempo de internação hospitalar, dias	33 (22-55)¶	35 (28-61)¶	30 (16-49)¶	0,024‡
Pacientes que não receberam TRS		1.145 (100,0)	1.019/1.113 (91,6)	94/1.113 (8,4)	
	Tempo de internação em UTI, dias	7 (3-13)	6 (3-11)	13 (6-17)	<0,001‡
	Tempo de internação hospitalar, dias	12 (8-19)	11 (8-19)	15 (9-24)	0,009‡
De acordo com o uso de ECMO*					
	Pacientes que receberam ECMO	20 (100,0)	6/17 (35,3)	11/17 (64,7)&	
	Tempo de internação em UTI, dias	32 (24-59)&	37 (23-76)&	29 (10-55)#	0,687‡
	Tempo de internação hospitalar, dias	48 (29-70)&	49 (33-98)&	31 (12-70)	0,365‡
Pacientes que não receberam ECMO		1.276 (100,0)	1.072/1.231 (87,1)	159/1.231 (12,9)	
	Tempo de internação em UTI, dias	7 (3-15)	7 (3-13)	15 (9-28)	<0,001‡
	Tempo de internação hospitalar, dias	12 (8-23)	12 (8-21)	18 (12-34)	<0,001‡

Resultados expressos como n/total n (%) ou mediana (intervalo interquartil).

* No momento da extração dos dados (9 de março de 2021), 48 pacientes ainda estavam hospitalizados. Destes, 36 pacientes estavam recebendo VM, 16 TRS e 3 ECMO; Valores de p foram calculados usando

† teste do χ^2^

‡ teste U de Mann-Whitney U

§ p<0,001 *versus* pacientes que não receberam VM

¶ p<0,001 *versus* pacientes que não receberam TRS

& p<0,001 *versus* pacientes que não receberam ECMO

# p=0,039 *versus* pacientes que não receberam ECMO.

UTI: unidade de terapia intensiva; VM: ventilação mecânica; TRS: terapia renal substitutiva; ECMO: oxigenação por membrana extracorpórea.

**Figura 1:** Tempo de internação em unidade de terapia intensiva e hospitalar por mês, mortalidade em unidade de terapia intensiva e hospitalar e escore *Simplified Acute Physiology* 3, de março de 2020 a fevereiro de 2021

**Figure 2:** Sobrevida cumulativa de 28 dias de todos os pacientes e de acordo com a necessidade de terapia de suporte

A mediana IIQ do tempo de internação em UTI e hospitalar foi de, respectivamente, 7 (4 a 16) dias e 13 (8 a 23) dias. Em comparação aos sobreviventes, os não sobreviventes tiveram tempo mais longo de internação em UTI [15 (9-29) *versus* 7 (3-13) dias; p<0,001] e hospitalar [19 (12-34) *versus* 12 (8-21) dias; p<0,001] (Tabela 3).

#### Pacientes em ventilação mecânica

Os pacientes que usaram ventilação mecânica tiveram mortalidade hospitalar mais alta em comparação àqueles que não a usaram (34,1% *versus* 4,3%; RC não ajustada de 11,5; IC95%: 7,8-17,0; p<0,001). Os tempos de internação em UTI [20 (13-32) *versus* 5 (2-8) dias; p<0,001] e hospitalar [27 (17-41) *versus* 10 (7-15) dias; p<0,001] foram mais longos em pacientes que usaram ventilação mecânica em comparação aos que não usaram ventilação mecânica.

#### Pacientes que receberam terapia renal substitutiva

Os pacientes que receberam TRS tiveram maior mortalidade hospitalar em comparação àqueles que não a receberam (56,3% *versus* 8,4%; RC não ajustada, 14,0; IC95%: 9,4-20,8; p<0,001). Ventilação mecânica foi necessária em 143 de 151 (94,7%) pacientes que receberam TRS. Os tempos de internação em UTI [27 (15-40) *versus* 7 (3-13) dias; p<0,001] e hospitalar [33 (22-55) *versus* 12 (8-19) dias; p<0,001] foram mais longos entre pacientes que receberam TRS do que naqueles que não receberam TRS.

#### Pacientes que receberam oxigenação por membrana extracorpórea

Os pacientes que usaram ECMO tiveram mortalidade hospitalar mais alta comparados ao que não receberam ECMO [64,7% *versus* 12,9%; RC não ajustada 12,4; IC95%: 4,5-33,9; p<0,001) (Tabela 3). Todos os pacientes que usaram ECMO receberam ventilação mecânica, e 13 de 20 (65,0%) pacientes também receberam TRS. Os tempos de internação em UTI [32 (24-59) *versus* 7 (3-15) dias; p<0,001] e hospitalar [48 (29-70) *versus* 12 (8-23) dias; p<0,001] foram mais longos nos pacientes que usaram ECMO do que naqueles que não usaram.

#### Preditores de mortalidade hospitalar

A análise univariada dos fatores associados à mortalidade hospitalar está descrita na tabela 4. Após ajuste para fatores de confusão, os preditores independentes de mortalidade hospitalar incluíram idade (RC: 1,08; IC95%: 1,06-1,10; p<0,001); SOFA (RC: 1,18; IC95%: 1,08-1,29; p<0,001); Índice de Comorbidade de Charlson (RC: 1,28; IC95%: 1,15-1,43; p<0,001); necessidade de ventilação mecânica (RC: 4,45; IC95%: 2,43-8,16; p<0,001); necessidade de CNAF (RC: 1,64; IC95%: 1,04-2,58; p=0,033); TRS (RC: 3,42; IC95%: 1,96-5,98; p<0,001) e suporte de ECMO (RC: 8,18; IC95%: 2,48-27,05; p<0,001) (Tabela 4). O modelo multivariado final apresentou ASC Característica de Operação do Receptor (IC95%) de 0,93 (0,91-0,94) e Hosmer-Lemeshow χ^2^ de 8.844 (p=0,356).

**Tabela 4:** Regressão logística univariada e multivariada de fatores de risco para mortalidade hospitalar

Preditores		Tipo de análise Análise univariada			Análise multivariada		
		RC	IC95%	Valor de p	RC	IC95%	Valor de p
Idade, anos		1,07	1,06-1,08	<0,001	1,08	1,06-1,10	<0,001
SAPS 3*							
	≤42	Referência					
	43-49	2,93	1,04-8,23	0,041			
	50-55	9,77	3,75-25,46	<0,001			
	≥56	41,80	16,79-104,07	<0,001			
SOFA		1,46	1,38-1,54	<0,001	1,18	1,08-1,29	<0,001
Índice de Comorbidade de Charlson		1,39	1,29-1,51	<0,001	1,28	1,15-1,43	<0,001
MFI		1,72	1,53-1,95	<0,001			
Comorbidade							
	Câncer	2,03	1,19-3,46	0,009			
	Insuficiência cardíaca congestiva	3,00	1,79-5,05	<0,001			
	DPOC	1,70	0,95-3,01	0,072			
	Doença renal crônica	2,83	1,53-5,22	<0,001			
	Doença renal crônica com necessidade de TRS	3,99	1,46-10,87	0,007			
	Câncer hematológico	2,10	1,02-4,31	0,045			
	Câncer metastático	2,38	0,95-5,92	0,063			
Dias de internação até a admissão em UTI		1,00	0,99-1,02	0,534			
LRA no momento da admissão na UTI		3,41	1,86-6,26	<0,001			
Suporte durante a internação em UTI							
	Ventilação mecânica	11,48	7,77-16,97	<0,001	4,45	2,43-8,16	<0,001
	Vasopressores	9,89	6,79-14,40	<0,001			
	Cateter nasal de alto fluxo	1,84	1,32-2,56	<0,001	1,64	1,04-2,58	0,033
	Terapia renal substitutiva	11,96	9,36-20,84	<0,001	3,42	1,96-5,98	<0,001
	ECMO	12,36	4,51-33,89	<0,001	8,18	2,48-27,05	<0,001

* O SAPS 3 foi categorizado de acordo com os percentis, já que o pressuposto de linearidade foi quebrado. O modelo multivariado apresentou área sob a curva Característica de Operação do Receptor (IC95%) de 0,93 (0,91-0,94) e Hosmer-Lemeshow χ^2^ de 8.844 (p=0,356).

RC: razão de chance; IC95%: intervalo de confiança de 95%; SAPS 3: *Simplified Acute Physiology Score 3*; SOFA: *Sequential Organ Failure Assessment Score*; MFI: *modified frailty index*; DPOC: doença pulmonar obstrutiva crônica; TRS: terapia renal substitutiva; UTI: unidade de terapia intensiva; LRA: lesão renal aguda; ECMO: oxigenação por membrana extracorpórea.

## DISCUSSÃO

Neste estudo de coorte retrospectivo em centro único, identificou-se que um em cada sete pacientes admitidos em UTI por quadro grave da COVID-19 morreu no hospital. Os não sobreviventes eram mais idosos; tinham quadro mais grave, com base no SAPS 3 e no SOFA; tinham mais comorbidades, como câncer, insuficiência cardíaca congestiva ou doença renal crônica, e maior tempo de internação em UTI e hospitalar, em comparação aos sobreviventes. Finalmente, idade mais avançada, pontuação mais alta no SOFA e no índice de comorbidade de Charlson, necessidade de ventilação mecânica, CNAF, TRS e ECMO foram preditores independentes de mortalidade hospitalar.

A associação entre idade avançada e maior risco de morte em pacientes infectados pelo SARS-CoV-2 já foi relatada por diferentes autores.^(3,26-28)^ Além disso, a associação entre a presença de comorbidades e a gravidade da COVID-19 foi comprovada em diversos estudos.^(9,26,29,30)^ Por exemplo, pacientes com COVID-19 e com hipertensão, doenças cardiocerebrovasculares e *diabetes mellitus* tiveram mais risco de apresentar sintomas graves e precisar de cuidados intensivos do que pacientes sem essas comorbidades.^(30)^ Também diagnóstico de câncer locorregional foi mais prevalente nos não sobreviventes em comparação aos sobreviventes. De fato, foi demonstrado, em um grande estudo de caso-controle, que pacientes com câncer com COVID-19 têm maior risco de piores prognósticos clínicos.^(31)^ Embora maior mortalidade já tenha sido registrada em pacientes com câncer hematológico,^(32)^ essa associação não foi constatada neste estudo.

Curiosamente, nesta coorte, a mortalidade hospitalar foi mais baixa, tanto entre os pacientes admitidos na UTI quanto entre aqueles que usaram ventilação mecânica, em comparação a outros estudos conduzidos no Brasil^(14)^ e em outros países.^(6,33,34)^ Em estudo de coorte envolvendo 254.288 pacientes hospitalizados com COVID-9 no Brasil, Ranzani et al., demonstraram taxa de mortalidade hospitalar de aproximadamente 60% entre os pacientes admitidos na UTI e de cerca de 80% entre aqueles que receberam ventilação mecânica.^(14)^ Em uma metanálise de um total de 28 estudos e 12.437 pacientes admitidos na UTI com COVID-19 em todo o mundo, a mortalidade dos pacientes admitidos em UTI e mecanicamente ventilados foi de 28,3% e 43%, respectivamente.^(4)^

A discrepância entre a taxa de mortalidade observada neste estudo e em outras casuísticas pode ser explicada por diferenças nos hospitais (privados comparados aos públicos); limiares adotados para internação em hospital e/ou UTI; disponibilidade de recursos, como leitos de UTI, e oferta limitada de suporte respiratório avançado fora da UTI, ou em UTIs com recursos limitados; características da equipe de UTI; estratégias de ventilação, como uso de ventilação não invasiva ou CNAF, e estratégias para suporte extrapulmonar. Além disso, apesar dos diferentes momentos da pandemia da COVID-19 no Brasil, a estabilidade mensal observada nos desfechos (tempo de internação e mortalidade) de pacientes da COVID-19 reflete a organização dos hospitais e das UTIs para oferecer a melhor qualidade de atendimento aos pacientes.

Pacientes com lesão renal aguda (LRA) que receberam TRS tiveram aumento de 14 vezes no risco de mortalidade hospitalar, comparados aos pacientes da COVID-19 que não receberam TRS. Essa observação condiz com a de outros estudos, em que foi relatada associação significativa entre insuficiência renal aguda e maior risco de mortalidade em pacientes com quadro grave da COVID-19.^(4,35,36)^ Além disso, quase metade dos pacientes não sobreviventes recebeu TRS, o que também se assemelha a outros estudos.^(34,37,38)^ A avaliação dos fatores de risco para LRA e o uso de TRS nos primeiros 207 pacientes graves da COVID-19 admitidos nesta UTI foram descritos em outras publicações.^(39)^

A maior taxa de mortalidade hospitalar neste estudo ocorreu no subgrupo de pacientes que recebeu ECMO (aproximadamente 65%). A mortalidade registrada entre pacientes da COVID-19 submetidos à ECMO venovenosa variou amplamente.^(40)^ Por exemplo: a mortalidade hospitalar registrada 90 dias após o início da ECMO em 1.035 pacientes do registro *Extracorporeal Life Support Organization* (ELSO) foi de 37%.^(41)^ É importante ressaltar que aproximadamente um terço dos pacientes incluídos neste estudo ainda estava hospitalizado ou tinha sido transferido para outro hospital ou para um centro de cuidados de longo prazo no momento em que os desfechos foram avaliados.^(41)^ Portanto, a mortalidade bruta dos pacientes da COVID-19 que receberam ECMO pode ter sido subestimada.

Este estudo tem limitações. Primeiro, foi realizado em uma única UTI, localizada em um hospital quaternário privado no Brasil. Portanto, os resultados podem não ser generalizáveis para outras UTIs do Brasil ou de outros países em desenvolvimento, porque os sistemas de saúde e as características dos pacientes podem variar amplamente em relação às da presente coorte. Segundo, não foram coletados dados detalhados sobre estratégias de ventilação não invasiva ou invasiva. Já foi comprovado que o suporte ventilatório em pacientes com IRA tem impacto importante nos desfechos.^(42,43)^ Terceiro, pressupõe-se que pacientes que receberam alta hospitalar antes de 28 dias ainda estariam vivos no dia 28. Entretanto, após a alta, esses pacientes podem ter sido reinternados em outro estabelecimento ou morrido após a alta. Finalmente, não se avaliaram se as novas variantes do SARS-CoV-2 influenciaram nos desfechos desta coorte de pacientes, em comparação às variantes que já circulavam no Brasil.^(44,45)^

## CONCLUSÃO

Pacientes com quadro grave da COVID-19 admitidos na unidade de terapia intensiva apresentaram considerável mortalidade e morbidade, com alta demanda de terapia de suporte e internação prolongada unidade de terapia intensiva e hospitalar. O volume e a gravidade dos pacientes da COVID-19 admitidos em unidade de terapia intensiva representam grande ônus para o sistema de saúde brasileiro. Portanto, os resultados deste estudo podem ser usados como referência, ou em apoio a decisões relativas à prestação de serviços de saúde e prognóstico de pacientes da COVID-19 que necessitem de cuidados intensivos.
